# Synchronous Laparoscopic Radical Nephrectomy Left and Contralateral Right Hemicolectomy during the Same Endoscopic Procedure

**DOI:** 10.5402/2011/179456

**Published:** 2011-04-26

**Authors:** G. M. Veenstra, L. M. C. L. Fossion, K. de Laet, A. A. P. M. Luijten

**Affiliations:** ^1^Faculty of Health, Medicine and Life Sciences, Maastricht University, 6200 MD Maastricht, The Netherlands; ^2^Maxima Medisch Centrum, De Run 4600, 5504 DB Veldhoven, The Netherlands

## Abstract

Synchronous renal cell carcinoma in patients with colorectal carcinoma is reported in various percentages ranging from 0.03 up to 4.85% (Halak et al. (2000), Capra et al. (2003)). When surgical treatment is indicated usually two separate operations are planned for resection. In open surgery, in such cases simultaneous resection is recommended if possible. Few reports have described the resection of colorectal and renal cell carcinoma in a single laparoscopic procedure. We have shown that combining left radical nephrectomy and right hemicolectomy is technically feasible, safe and that overall operative time can be limited. In our case operative time was 210 minutes, blood loss 100 milliliters, and duration of hospital stay was 8 days. Adequate port placement, preoperative scheduling, and surgical experience are essential to achieve this goal.

## 1. Introduction

Primary cancer may occur synchronously in different organs. Synchronous renal cell carcinoma in patients with colorectal carcinoma is reported in various percentages ranging from 0.03 to 4.85% [1, 2]. When surgical treatment is indicated, usually two separate operations are planned for resection. Since the successful introduction of laparoscopic colectomy by Jacobs et al. [3], laparoscopic surgery for the treatment of colorectal cancer has been developed considerably. For the treatment of localized renal cell carcinoma, laparoscopic radical nephrectomy is now the golden standard when partial resection is not indicated [4]. Few reports have described a simultaneous laparoscopic nephrectomy and ipsilateral hemicolectomy [5–9]. To our knowledge this is the first report about a synchronous laparoscopic left radical nephrectomy left with contralateral laparoscopic hemicolectomy with a extracorporeal anastomosis.

## 2. Case Report

**Figure 1:**
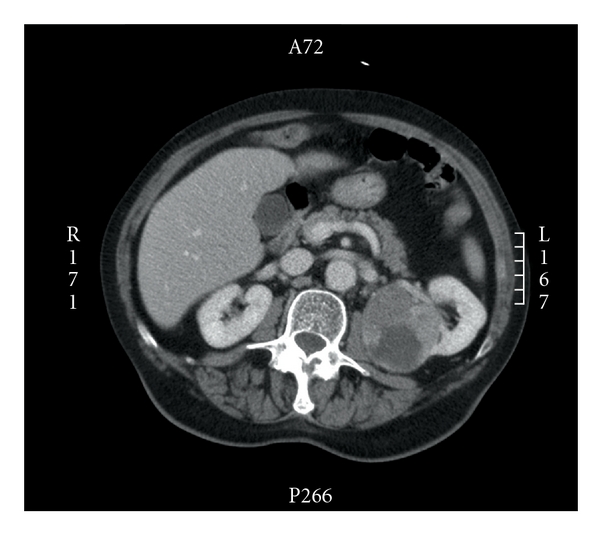
CT transversal image showing a 7 cm tumor in the upper pole of the left kidney with central necrosis.

**Figure 2:**
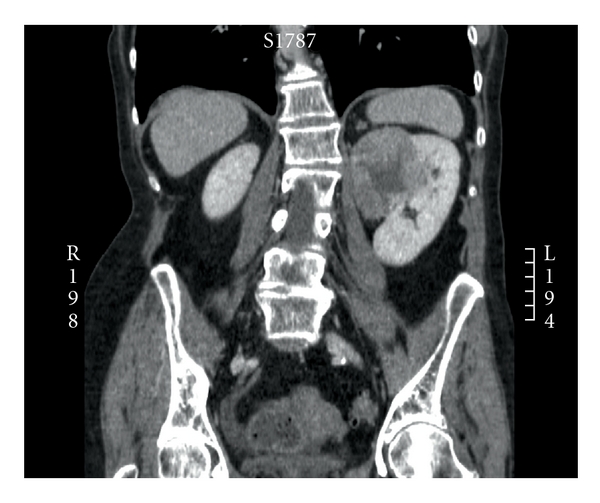
CT coronal image of the kidney tumor. The upper pole tumor lies medially in the left kidney.

**Figure 3:**
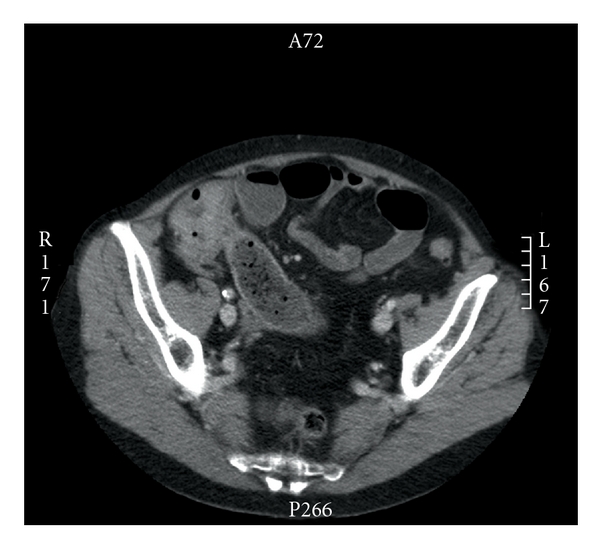
CT transversal image showing a tumor of the colon ascendens.

A 70-year-old female was admitted to the emergency unit with a tingling in the right hand, dysphasia, and progressive abdominal pain. Physical examination revealed no major abnormalities. Blood results showed an anemia with hemoglobin of 4.6 mmol/L (norm > 7.5 mmol/L). A CT-cerebrum showed no signs of bleeding, infarction, or metastasis of the brain. Abdominal CT showed a tumor of the left kidney and right colon ascendens with lymphadenopathy (Figures 1, 2, and 3). No active bleeding was suspected. One week later a colonoscopy was performed confirming a circular growing tumor in the colon ascendens. Biopsies showed a well-differentiated colon adenocarcinoma. The patient subsequently underwent synchronous laparoscopic resection of the renal tumor and the colon tumor within three weeks after initial admission. Patient was classified with an ASA score of III [10].

## 3. The Procedure

**Figure 4:**
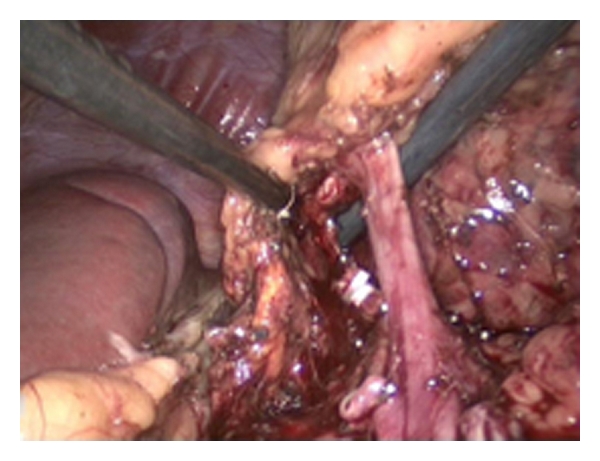
The arteria renalis has been clipped. The vena renalis is shown at the right, ready to be clipped. The spleen is visible on the left side.

**Figure 5:**
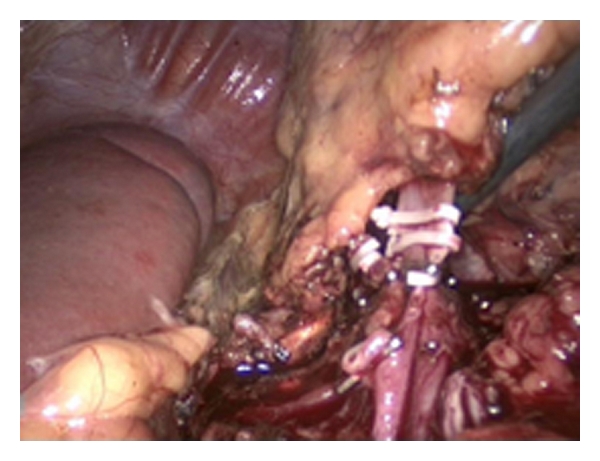
The vena renalis has been clipped and is ready to be cut.

The patient was placed in the right lumbotomy position. The urologist performed the left transperitoneal radical nephrectomy. The first trocar was placed in a paraumbilical way through the open introduction technique according to Hasson [11]. The pneumoperitoneum was established through this 12 mm, port, and a pressure of 15 mmHg was maintained. One additional 10-mm, and one 5-mm trocar were then inserted under laparoscopic vision in the epigastric and midclaviculair position. Port placement for the radical nephrectomy was not in an altered configuration. The colon was reflected medially by dissecting the Toldt fascia, exposing the anterior aspect of the left kidney. The ureter was followed to the hilum of the kidney to safely approach the vessels. Renal artery and vein were separately clipped with Hem-o-locks (see Figures 4 and 5). The kidney with adrenal gland, perirenal fat tissue, and surrounding Gerota's fascia was dissected and bloc and positioned inside an endoscopic bag. 

An abdominal surgeon performed the next part of the operation. The patient's position was changed to supine. The surgeon used the previously placed periumbilical and epigastric 12 mm ports. One additional 5 mm trocar was placed. A slightly altered port configuration was used. The camera was used in the periumbilical port as well as in the more cranial epigastric port. Using the harmonic scalpel and a bowel clamp, the right paracolic gutter was detached, starting from the corner of the caecum towards the liver and the gallbladder. The ileocolic artery was dissected and secured with Hem-o-lok clips. The transverse colon was cut using a TLC stapler. The terminal ileum was cut with staplers. The mesentery was cut with Ultracision. The subumbilical port site was enlarged to remove the colon and the endobag containing the left kidney. A handsewn side-to-side anastomosis between the terminal ileum and transverse colon was performed with continuous PDS 3/0. The mesenteric defect was closed with separate stitches PDS 3/0. The colon was then replaced in the abdomen, and the paraumbilical incision was closed with a continuous Vicryl suture. A left paracolic drain was left in the renal lodge, trocars were removed under vision, and wounds were closed in layers.

Total operation time and blood loss were 210 minutes and 100 milliliter. The operation time needed for laparoscopic tumor nephrectomy was 110 minutes, and blood loss was 50 milliliter. For the hemicolectomy, operative time and blood loss were 100 minutes and 50 milliliter, respectively. The procedure was uncomplicated. On postoperative day 2 the wound drain was removed, and on day 4 the patient had flatus and the first bowel motion. The patient was discharged 8 days postoperatively. There were no postoperative complications. 

Pathological examination showed a kidney of 120 × 60 × 35 mm. containing a clear cell renal cell carcinoma Fuhrman grade 4 with partly sarcomatoid growth with a maximum diameter of 75 mm in the upper renal pole. The tumor was confined within the renal capsule. The adrenal gland was free of tumor. The right hemicolectomy specimen revealed a moderately differentiated adenocarcinoma of 4 cm with exophytic growth and invasion through the muscularis propria into the surrounding tissue, but not into the serosa. One of the twelve lymph nodes showed metastasis. All resection margins were free in both procedures. Pathologic classification according to the TNM classification showed a renal cell tumor, pT3aNxMx clear cell and Fuhrman grade IV and colon adenocarcinoma pT3N1/12Mx.

Adjuvant chemotherapy was started with oxaliplatin and capecitabine. Followup after 9 months showed no metastasis on CT-abdomen and a carcinoembryonic antigen (CEA) level of 2.7 *μ*g/L, norm < 3.0. Unfortunately, our patient was diagnosed with cerebral and pulmonary metastases 10 months after surgery. Biopsy showed metastasis of adenocarcinoma. She was treated with Gamma Knife radiosurgery and palliative chemotherapy.

## 4. Discussion

This case report confirms that left radical nephrectomy and right hemicolectomy can be combined in one laparoscopic procedure. Previously described reports in the literature reported only synchronous renal and colon resection on the same side of the abdomen [5–9]. Thus this report shows the feasibility of combining a left-sided and a right-sided laparoscopic procedure. We have seen some advantages of the laparoscopic approach with regard to postoperative pain, hospitalization time, return to normal activities, and cosmetically acceptable incisions. In our case operative time was 210 minutes, blood loss 100 milliliters, and duration of hospital stay was 8 days. The operation time during this laparoscopic procedure was short and comparable with open procedures. However, 210 minutes is longer than a single laparoscopic procedure. Experience and completing the procedure in time may play a role in avoiding complications and result in good oncologic results. Despite our unfortunate oncological outcome we have no reason to blame the synchronous approach. No port site metastasis was found, and resection was en bloc with adequate resection margins. Nevertheless more research has to be done for oncological followup in the future with synchronous tumor resections. When possible, we still recommend a synchronous laparoscopic procedure for a double tumor in kidney and colon. Such procedure requires adequate port placement and a multidisciplinary approach with good operation scheduling. A limitation of the procedure is that completing the operation safely, surgical experience in advanced laparoscopic techniques is required.

## Figures and Tables

**Figure 6:**
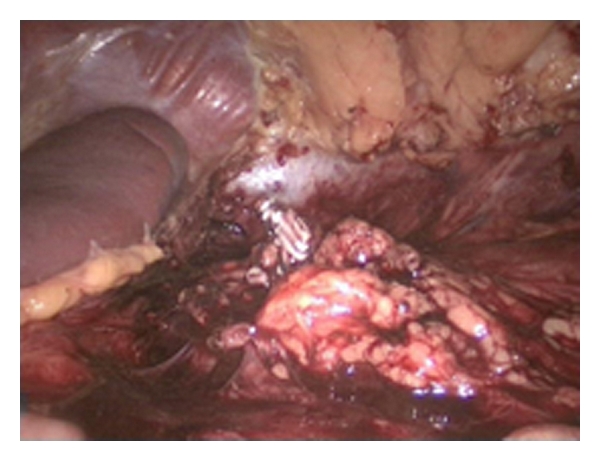
Inspection of the renal lodge at the end of the procedure.
